# Effect of Exercise on Motor and Nonmotor Symptoms of Parkinson's Disease

**DOI:** 10.1155/2015/586378

**Published:** 2015-02-02

**Authors:** Khashayar Dashtipour, Eric Johnson, Camellia Kani, Kayvan Kani, Ehsan Hadi, Mark Ghamsary, Shant Pezeshkian, Jack J. Chen

**Affiliations:** ^1^Department of Neurology, School of Medicine, Loma Linda University, Loma Linda, CA 92354, USA; ^2^Department of Physical Therapy, School of Allied Health Professions, Loma Linda University, Loma Linda, CA 92354, USA; ^3^Department of Epidemiology and Biostatistics, School of Public Health, Loma Linda University, Loma Linda, CA 92354, USA; ^4^Department of Pharmacy Practice, College of Pharmacy, Marshal B. Ketchum University, Fullerton, CA 92831, USA

## Abstract

*Background*. Novel rehabilitation strategies have demonstrated potential benefits for motor and non-motor symptoms of Parkinson's disease (PD).* Objective*. To compare the effects of Lee Silverman Voice Therapy BIG (LSVT BIG therapy) versus a general exercise program (combined treadmill plus seated trunk and limb exercises) on motor and non-motor symptoms of PD.* Methods*. Eleven patients with early-mid stage PD participated in the prospective, double-blinded, randomized clinical trial. Both groups received 16 one-hour supervised training sessions over 4 weeks. Outcome measures included the Unified Parkinson's Disease Rating Scale (UPDRS), Beck Depression Inventory (BDI), Beck Anxiety Inventory (BAI) and Modified Fatigue Impact Scale (MFIS). Five patients performed general exercise and six patients performed LSVT BIG therapy. Post-intervention evaluations were conducted at weeks 4, 12 and 24.* Results*. The combined cohort made improvements at all follow-up evaluations with statistical significance for UPDRS total and motor, BDI, and MFIS (*P* < 0.05).* Conclusion*. This study demonstrated positive effects of general exercise and LSVT BIG therapy on motor and non-motor symptoms of patients with PD. Our results suggest that general exercise may be as effective as LSVT BIG therapy on symptoms of PD for patients not able to readily access outpatient LSVT BIG therapy.

## 1. Introduction

Parkinson's disease (PD) is a neurodegenerative disorder consisting of a constellation of motor and nonmotor symptoms. For many years, even before the availability of medical therapies for PD, clinicians have been trying physical activities to improve symptoms of PD. In recent years, basic science evidence based on animal models suggests that the effects of the exercise are beyond solely symptomatic therapies [1–4]. In 1999, van Praag et al. showed the increase of neurogenesis following exercise in the adult brain rats [5]. Since then, other scientists have replicated this finding and clinicians have tried to assess and quantify the effect of exercise on a variety of neurological disorders [6, 7]. Multiple clinical studies demonstrated the benefit of exercise on motor symptoms of PD by applying different exercise models such as treadmill training [8–10], resistance training [11–13], biking [14], Tai Chi [15, 16], tango [17, 18], and boxing [19]. One of the exercise models that has been shown to be effective and is currently being used widely is Lee Silverman Voice Therapy BIG (LSVT BIG therapy) [20–22]. LSVT BIG therapy is designed to overcome amplitude deficits associated with PD. This therapy improves proprioception through increasing amplitude together with sustained attention and cognitive involvement by mentally focusing on individual movements. Emerging evidence examining the benefits of LSVT BIG therapy for patients with PD has demonstrated improvements in gait speed and the speed of reaching across the upper and lower limbs as well as improvements of the UPDRS motor score [21, 22].

LSVT BIG therapy incorporates large trunk and extremity functional motions and should only be administered by an LSVT BIG certified therapist. In contrast to other conventional training methods that use various techniques to facilitate movements, the LSVT BIG therapy uses amplitude as the main focus of the exercise and engages the patient in functional activities with the promise of sustainability after completion of the therapy [23].

Recent studies have provided evidence that exercise modalities can improve motor and nonmotor features of PD [24–26]. However, the effects of LSVT BIG therapy on nonmotor symptoms of PD have not yet been extensively studied. The purpose of this study was to determine the difference between two various exercise programs, namely, the LSVT BIG therapy and a general exercise program, on both motor and nonmotor symptoms of PD. To our knowledge, this is the first study comparing a general exercise modality with LSVT BIG therapy in a double-blinded prospective trial.

## 2. Materials and Methods

### 2.1. Participants

Eleven patients with early- to mid-stage PD participated in a prospective, double-blinded, randomized study. All patients were recruited from the movement disorders clinic at the Loma Linda University Medical Center following the Institutional Review Board approval and obtaining informed consent. PD was diagnosed according to the UK Parkinson Disease Society Brain Bank Criteria [27]. Patients were included if they were aged 30 to 90 years, if they were on a stable dose of PD medications for the last 28 days, if their clinical condition at the time of study enrollment did not require any changes of medication for the next four months, and if they were clinically stable to attend either the outpatient physical therapy for LSVT BIG therapy or a general exercise program at the Loma Linda University Research Laboratory for sixteen 1-hour sessions over four weeks. Exclusion criteria were diagnosis of atypical PD, participation in an ongoing exercise program, history of repeated strokes with stepwise progression of Parkinsonian features, evidence of severe depression or other significant behavioral disorders, significant or unstable medical or surgical condition that may preclude safe and complete study participation. Patients were randomly assigned to receive either general exercise (five patients) or LSVT BIG therapy (six patients).

### 2.2. Outcome Measures

All patients were evaluated on the Unified Parkinson's Disease Rating Scale (UPDRS) (total and motor scales), Beck Depression Inventory (BDI), Beck Anxiety Inventory (BAI), and Modified Fatigue Impact Scale (MFIS) at baseline, immediately upon completion of exercise program (after four weeks) and after three and six months' follow-up. Exercise treatments were administered by an experienced physical therapist four times a week for four weeks. Both intervention groups received 16 one-hour one to one exercise sessions. After completion of exercise training, the patients in both interventional groups were advised to maintain an active lifestyle and exercise on a regular basis.

### 2.3. Interventions

#### 2.3.1. General Exercise Protocol

The General Exercise protocol consisted of two parts: a thirty-minute treadmill exercise session and a seated upper extremity exercise session. During the treadmill exercise session, patients walked on a treadmill continuously for 30 minutes with the following criteria: they used a safety harness that was attached to a sturdy frame. A safety cord was attached to the patients that would quickly stop the treadmill if necessary. A research investigator was positioned next to the patient at all times. The angle of inclination of the treadmill was zero degree. Walking speed was gradually increased until the patient was walking comfortably and reported an exertion level of approximately five on a ten-point Borg rating of perceived exertion scale [28]. The Borg scale was displayed in front of the patient at all times during treadmill walking. Heart rate was monitored while walking using a wireless heart rate monitor. Patients were working at 75% of their heart rate maximum. Heart rate maximum was calculated using the Karvonen formula prior to commencement of the session. Blood pressure (BP) was monitored before, during, and immediately after the treadmill exercise session. A systolic BP of less than 200 mm Hg and diastolic BP of less than 110 mm Hg were considered within safe limits during the treadmill exercise session. Afterward, systolic BP was less than 180 mm Hg and diastolic BP was less than 110 mm Hg before beginning the seated upper extremity exercise session.

During the seated upper extremity exercise session, patients performed a variety of upper extremity exercises for 30 minutes. Exercise intensity was monitored by the Borg's scale. The seated upper extremity protocol included the following high to low intensity exercises: theraband arm swings for 4 minutes (high intensity, Borg 4-5), trunk side flexion for 2 minutes (medium intensity, Borg 3-4), marching in place for 2 minutes (medium intensity, Borg 3-4), buttoning/unbuttoning for 2 minutes (low intensity, Borg 2-3), theraband rowing for 4 minutes (high intensity, Borg 4-5), trunk rotations with reaching for 2 minutes (medium intensity, Borg 3-4), cone stacking for 2 minutes (medium intensity, Borg 3-4), finger tapping for 2 minutes (low intensity, Borg 2-3), theraband trunk rotation for 4 minutes (high intensity, Borg 4-5), marching in the place with arm swings for 2 minutes (high intensity, Borg 4-5), seated arm swings for 2 minutes (medium intensity, Borg 3-4), and finger to nose for 2 minutes (low intensity, Borg 2-3).

#### 2.3.2. LSVT BIG Therapy

The LSVT BIG therapy was delivered by an LSVT BIG certified physical therapist. The LSVT BIG therapy protocol has been previously described and patients were essentially encouraged to perform a variety of large amplitude functional movements with high concentration and effort over the course of 60 minutes with supervision [20].

### 2.4. Data Analyses

SAS Version 9.3 (SAS Institute Inc., Cary, NC) was used for statistical analysis. Descriptive statistics (mean, standard deviation) were calculated for all assessments. An independent* t*-test was used to analyze normally distributed baseline differences between the groups. For non-normally distributed data, Wilcoxon's rank-sum test (Mann-Whitney U test) was used. Paired samples* t*-test with its nonparametric version (Wilcoxon signed rank test) was used for dependent samples. Changes in the test scores (UPDRS total, UPDRS motor subscale, BDI, BAI, and MFIS) with exercise intervention, at the different time points (follow-up evaluations) were analyzed using ANOVA repeated measures design. The effects for each group (general exercise versus LSVT BIG therapy) across time were assessed using the similar set of analyses. A further permutation test was also used, because of small sample size in comparison groups. The* P*-value was calculated, and the significance was assumed as *p* less than or equal to 0.05.

## 3. Results

Table 1 shows the demographics and baseline characteristics in the two intervention groups. The mean age was 63.4 years. The female to male ratio was 6 : 5. The mean number of years since diagnosis was 3.8 (SD = 2.4). There were no statistically significant differences between the intervention groups on demographic or clinical characteristics at baseline.

All patients completed follow-up visits. Complete baseline and follow-up data were available for all patients. No major adverse events were reported during this trial, and there were no changes of medications related to PD.

Table 2 shows the clinical changes from the baseline in all eleven patients in both groups after exercise interventions.

Table 3 shows the benefit of general exercise and LSVT BIG therapy in each group at 6 months' follow-up. No difference was detected between the two groups.

## 4. Discussion 

The current double-blinded prospective trial showed the impact of LSVT BIG therapy on motor and nonmotor symptoms of PD and compared its impact with general exercise.

### 4.1. Impact of Exercise

The impact of exercise on PD already has been shown in different studies [24–26]. In the current trial, we simulated the one to one training with similar time and interaction of LSVT BIG therapy with a general exercise program. The positive impact of exercise and LSVT BIG therapy persisted for up to six months after the last day of the intervention and raised the possibility of disease modification that exercise might apply. There are bodies of evidence that support impact of the exercise on the brain with increasing synaptogenesis and neurotrophic factors [29, 30]. This trial was not able to detect the difference between the two groups but showed that our general exercise protocol was as effective as LSVT BIG therapy which may prove beneficial for patients unable to access outpatient LSVT BIG therapy.

### 4.2. LSVT BIG Therapy

The effect of LSVT BIG therapy on motor symptoms of PD has been assessed in at least two major trials [22, 23]. In the present study, our findings suggest that LSVT BIG therapy could be effective on controlling nonmotor symptoms of PD such as depression, anxiety, and fatigue. In a previous trial [22] comparing the efficacy of LSVT BIG therapy and exercise, a bias was detected in favor of LSVT BIG therapy. LSVT BIG therapy was conducted in a one-to-one fashion but exercise was applied in a group session. In the current study, to avoid this error, we designed a general exercise protocol that incorporated a one-to-one interaction between patients and their therapists.

### 4.3. Motor and Nonmotor Symptoms of PD

The main focus of the majority of previous research was on the motor aspect of PD such as bradykinesia, rigidity, and gait. None of the studies that applied LSVT BIG therapy measured nonmotor symptoms of PD as one of their outcome measures [21–23]. In fact, nonmotor symptoms are very disabling in patients with PD because the majority of them do not respond easily to medical therapy [31]. This emphasizes the importance of therapeutic modalities other than medications in managing nonmotor symptoms such as fatigue, depression, and anxiety.

The current study suggested that not only did both types of exercise improve motor symptoms of PD but also it was effective on nonmotor symptoms as well.

### 4.4. Limitations

Small sample size is one of the limitations of this study. All of the patients were at the early stage of their disease; therefore, the impact of the exercise on the symptoms of the patients at the later stages of the disease is unclear. With the progression of PD, patients develop certain motor and nonmotor complications such as motor fluctuation, dyskinesia, cognitive decline, gait impairment, and poor balance. None of the patients of this study were suffering from any of these complications.

## 5. Conclusion 

The results of the current study suggest that exercise is an essential therapeutic modality to manage motor and nonmotor symptoms of PD. This study also highlights the importance of creating other exercise protocols based on the LSVT BIG therapy that could be as effective for patients that do not have access to outpatient LSVT BIG therapy. Larger studies with more patients at different stages of the disease with multiple therapeutic arms are needed. The new trials would not only improve our understanding about the impact of exercise on neurodegenerative disorders, but also help us to improve our skills in managing patients with PD with multiple complications.

## Figures and Tables

**Table 1:** Demographic and clinical characteristics at baseline.

	General exercise (*N* = 5) mean (SD)	LSVT BIG therapy (*N* = 6) mean (SD)	*P* value
Age	64.0 (4.2)	62.8 (13.9)	0.85
Years since diagnosis	4.5 (3.3)	2.9 (1.5)	0.40
Hoehn and Yahr stage	1.3 (0.5)	1.8 (0.5)	0.21
UPDRS (total)	27.0 (10.2)	28.0 (8.1)	0.97
UPDRS (motor)	18.4 (5.6)	17.2 (5.8)	0.75
BDI	6.6 (2.3)	10.5 (4.8)	0.15
BAI	9.0 (5.2)	6.0 (4.7)	0.37
MFIS	15.6 (11.4)	27.7 (9.7)	0.18

**Table 2:** Clinical changes from baseline after exercise interventions in both groups (*N* = 11).

	Week 4 evaluation	*P* value	3-month evaluation	*P* value	6-month evaluation	*P* value
	Effect (SEM)		Effect (SEM)		Effect (SEM)	
ΔUPDRS (total)	−4.4 (2.6)	0.106	−3.4 (2.7)	0.226	−7.0 (2.7)	0.016
ΔUPDRS (motor)	−1.6 (1.8)	0.390	−2.6 (1.9)	0.176	−4.7 (1.9)	0.019
ΔBDI	−4.4 (0.9)	0.001	−3.3 (0.9)	0.002	−2.8 (0.9)	0.006
ΔBAI	−1.0 (1.4)	0.507	−1.7 (0.7)	0.031	−1.6 (1.3)	0.274
ΔMFIS	−4.2 (3.7)	0.264	−5.8 (3.8)	0.147	−8.9 (3.8)	0.030

**Table 3:** Comparison between the two intervention groups at 6-month evaluation.

	General exercise (*N* = 5)	LSVT BIG therapy (*N* = 6)	*P* value
	mean (SD)	mean (SD)	
ΔUPDRS	−5.0 (11.5)	−8.3 (11.0)	0.682
ΔUPDRS motor	−2.8 (9.6)	−6.8 (6.1)	0.503
ΔBDI	−2.0 (2.0)	−3.3 (1.5)	0.363
ΔBAI	−3.0 (5.6)	−0.5 (2.6)	0.440
ΔMFIS	−11.2 (12.8)	0.0 (1.4)	0.296

## References

[1] Petzinger G. M., Fisher B. E., van Leeuwen J.-E. (2010). Enhancing neuroplasticity in the basal ganglia: the role of exercise in Parkinson's disease. *Movement Disorders*.

[2] Tajiri N., Yasuhara T., Shingo T. (2010). Exercise exerts neuroprotective effects on Parkinson's disease model of rats. *Brain Research*.

[3] Tillerson J. L., Caudle W. M., Reverón M. E., Miller G. W. (2003). Exercise induces behavioral recovery and attenuates neurochemical deficits in rodent models of Parkinson's disease. *Neuroscience*.

[4] Fisher B. E., Petzinger G. M., Nixon K. (2004). Exercise-induced behavioral recovery and neuroplasticity in the 1-methyl-4-phenyl-1,2,3,6-tetrahydropyridine-lesioned mouse basal ganglia. *Journal of Neuroscience Research*.

[5] van Praag H., Kempermann G., Gage F. H. (1999). Running increases cell proliferation and neurogenesis in the adult mouse dentate gyrus. *Nature Neuroscience*.

[6] Lindsay J., Laurin D., Verreault R. (2002). Risk factors for Alzheimer's disease: a prospective analysis from the Canadian Study of Health and Aging. *American Journal of Epidemiology*.

[7] Chen H., Zhang S. M., Schwarzschild M. A., Hernán M. A., Ascherio A. (2005). Physical activity and the risk of Parkinson disease. *Neurology*.

[8] Fisher B. E., Wu A. D., Salem G. J. (2008). The effect of exercise training in improving motor performance and corticomotor excitability in people with early Parkinson's disease. *Archives of Physical Medicine and Rehabilitation*.

[9] Kurtais Y., Kutlay S., Tur B. S., Gok H., Akbostanci C. (2008). Does treadmill training improve lower-extremity tasks in Parkinson disease? A randomized controlled trial. *Clinical Journal of Sport Medicine*.

[10] Shulman L. M., Katzel L. I., Ivey F. M. (2013). Randomized clinical trial of 3 types of physical exercise for patients with parkinson disease. *JAMA Neurology*.

[11] Hass C. J., Buckley T. A., Pitsikoulis C., Barthelemy E. J. (2012). Progressive resistance training improves gait initiation in individuals with Parkinson's disease. *Gait and Posture*.

[12] Dibble L. E., Hale T. F., Marcus R. L., Gerber J. P., LaStayo P. C. (2009). High intensity eccentric resistance training decreases bradykinesia and improves quality of life in persons with Parkinson's disease: a preliminary study. *Parkinsonism & Related Disorders*.

[13] Corcos D. M., Robichaud J. A., David F. J. (2013). A two-year randomized controlled trial of progressive resistance exercise for Parkinson's disease. *Movement Disorders*.

[14] Alberts J. L., Linder S. M., Penko A. L., Lowe M. J., Phillips M. (2011). It is not about the bike, it is about the pedaling: forced exercise and Parkinson's disease. *Exercise and Sport Sciences Reviews*.

[15] Corcos D. M., Comella C. L., Goetz C. G. (2012). Tai chi for patients with Parkinson's disease. *The New England Journal of Medicine*.

[16] Li F., Harmer P., Fitzgerald K. (2012). Tai chi and postural stability in patients with Parkinson's disease. *The New England Journal of Medicine*.

[17] Hackney M. E., Earhart G. M. (2010). Effects of dance on balance and gait in severe Parkinson disease: a case study. *Disability and Rehabilitation*.

[18] Duncan R. P., Earhart G. M. (2012). Randomized controlled trial of community-based dancing to modify disease progression in Parkinson disease. *Neurorehabilitation and Neural Repair*.

[19] Combs S. A., Diehl D., Staples W. H. (2011). Boxing training for patients with parkinson disease: a case series. *Physical Therapy*.

[20] Farley B. G., Koshland G. F. (2005). Training BIG to move faster: the application of the speed-amplitude relation as a rehabilitation strategy for people with Parkinson's disease. *Experimental Brain Research*.

[21] Fox C. M., Raming L. O., Ciucci M. R., Sapir S., McFarland D. H., Farley B. G. (2006). The science and practice of LSVT/LOUD: neural plasticity—principled approach to treating individuals with parkinson disease and other nuerological disorders. *Seminars in Speech and Language*.

[22] Ebersbach G., Ebersbach A., Edler D. (2010). Comparing exercise in Parkinson's disease—the Berlin LSVTBIG study. *Movement Disorders*.

[23] Fox C., Ebersbach G., Ramig L., Sapir S. (2012). LSVT LOUD and LSVT BIG: behavioral treatment programs for speech and body movement in Parkinson disease. *Parkinson's Disease*.

[24] Herman T., Giladi N., Hausdorff J. M. (2009). Treadmill training for the treatment of gait disturbances in people with Parkinson's disease: a mini-review. *Journal of Neural Transmission*.

[25] Mehrholz J., Friis R., Kugler J., Twork S., Storch A., Pohl M. (2010). Treadmill training for patients with Parkinson's disease. *The Cochrane Database of Systematic Reviews*.

[26] Dibble L. E., Addison O., Papa E. (2009). The effects of exercise on balance in persons with parkinson's disease: a systematic review across the disability spectrum. *Journal of Neurologic Physical Therapy*.

[27] Hughes A. J., Ben-Shlomo Y., Daniel S. E., Lees A. J. (1992). What features improve the accuracy of clinical diagnosis in Parkinson's disease: a clinicopathologic study. *Neurology*.

[28] Borg G. A. V. (1982). Psychophysical bases of perceived exertion. *Medicine and Science in Sports and Exercise*.

[29] Fabel K., Kempermann G. (2008). Physical activity and the regulation of neurogenesis in the adult and aging brain. *NeuroMolecular Medicine*.

[30] Erickson K. I., Gildengers A. G., Butters M. A. (2013). Physical activity and brain plasticity in late adulthood. *Dialogues in Clinical Neuroscience*.

[31] Chaudhuri K. R., Healy D. G., Schapira A. H. V. (2006). Non-motor symptoms of Parkinson's disease: diagnosis and management. *Lancet Neurology*.

